# Multisystem Synthesis of Radar Sounding Observations of the Amundsen Sea Sector From the 2004–2005 Field Season

**DOI:** 10.1029/2021JF006296

**Published:** 2021-10-22

**Authors:** Winnie Chu, Andrew M. Hilger, Riley Culberg, Dustin M. Schroeder, Thomas M. Jordan, Helene Seroussi, Duncan A. Young, Donald D. Blankenship, David G. Vaughan

**Affiliations:** ^1^ School of Earth and Atmospheric Science Georgia Institute of Technology Atlanta GA USA; ^2^ Department of Electrical Engineering Stanford University Stanford CA USA; ^3^ Department of Geophysics Stanford University Stanford CA USA; ^4^ Plymouth Marine Laboratory Plymouth UK; ^5^ Thayer School of Engineering Dartmouth College Hanover NH USA; ^6^ Institute for Geophysics University of Texas at Austin Austin TX USA; ^7^ British Antarctic Survey Cambridge UK

## Abstract

The Amundsen Sea Embayment of the West Antarctic Ice Sheet contains Thwaites and Pine Island Glaciers, two of the most rapidly changing glaciers in Antarctica. To date, Pine Island and Thwaites Glaciers have only been observed by independent airborne radar sounding surveys, but a combined cross‐basin analysis that investigates the basal conditions across the Pine Island‐Thwaites Glaciers boundary has not been performed. Here, we combine two radar surveys and correct for their differences in system parameters to produce unified englacial attenuation and basal relative reflectivity maps spanning both Pine Island and Thwaites Glaciers. Relative reflectivities range from −24.8 to +37.4 dB with the highest values beneath fast‐flowing ice at the ice sheet margin. By comparing our reflectivity results with previously derived radar specularity and trailing bed echoes at Thwaites Glacier, we find a highly diverse subglacial landscape and hydrologic conditions that evolve along‐flow. Together, these findings highlight the potential for joint airborne radar analysis with ground‐based seismic and geomorphological observations to understand variations in the bed properties and cross‐catchment interactions of ice streams and outlet glaciers.

## Key Points

- We analyze two simultaneous 2004/2005 airborne radar surveys of Pine Island and Thwaites Glaciers conducted using different sounders
- We produce catchment‐wide englacial attenuation and basal reflectivity maps for Pine Island and Thwaites Glaciers
- Radar analysis reveals a critical frozen basal patch that currently separates the two neighboring glaciers

## 1 Introduction

The contribution of potentially unstable marine ice sheet sectors is one of the major sources of uncertainty in projections of sea‐level rise (IPCC, 2013). Bedrock topography and the spatial distribution of basal resistance beneath marine ice sheets determine the susceptibility of outlet glaciers to marine ice sheet instability, and thereby their contribution to sea level (Joughin et al., 2019; Koellner et al., 2019; Scambos et al., 2017). However, modeling ice sheet basal processes is challenging because basal conditions are difficult to characterize directly (Vaughan & Arthern, 2007).

Airborne radar echo sounding is one of the powerful geophysical methods used to investigate the subglacial environment at a catchment‐scale (Dowdeswell & Evans, 2004). Relative reflectivity can provide evidence for subglacial water based on its higher reflectivity values compared to drier bed regions (e.g., Chu, Schroedu, et al., 2016; Peters et al., 2005). Changes in reflectivity have also been used to infer variations in basal thermal state (Chu et al., 2018; MacGregor et al., 2016), basal roughness (e.g., Jordan et al., 2017), and bed lithology (e.g., Siegert et al., 2016). In addition, descriptions of bed echo character, such as specularity content and trailing bed echo (also known as cross‐track energy), enable further investigations on subglacial drainage geometry (e.g., Schroeder et al., 2013) and basal roughness (e.g., Young et al., 2016).

Despite these advances in radar sounding analysis and interpretation, very few studies combine data across multiple families of radar systems to assess bed characteristics (Winter et al., 2017) and different bed echo analysis techniques (Young et al., 2016) due to the challenges of system cross‐calibration and complications for joint interpretations of reflectivity and bed echo character. Nonetheless, having a coherent radar interpretation based on multiplatform observations and joint analysis of reflectivity and bed echo character will provide the critical baseline to develop a more complete understanding of the subglacial environment. In this study, we aim to perform this type of integrated radar analysis for the Amundsen Sea Embayment (ASE) of West Antarctica by leveraging two airborne radar sounding surveys.

The ASE contains both Pine Island Glacier (PIG) and Thwaites Glacier (TG), two of the most rapidly changing (Pritchard et al., 2009; Sutterley et al., 2014; Turner et al., 2016) and potentially unstable glaciers (Bamber et al., 2009; Favier et al., 2014; Joughin et al., 2014; Parizek et al., 2013; Seroussi et al., 2014) of the Antarctic ice sheet. Both glaciers are grounded on retrograde bed‐slopes (Favier et al., 2014; Joughin et al., 2014; Schoof, 2007) and their grounding lines have undergone rapid retreat in the last two decades (Milillo et al., 2019; Rignot et al., 2014). The primary trigger of this retreat is the incursion of warm modified Circumpolar Deep Water into the ice shelf cavities, driving increased sub‐ice‐shelf melting (e.g., Dutrieux et al., 2014; Jacobs et al., 2011). The subsequent thinning of the ice shelves reduces their back stress on the inland glaciers, which increased ice discharge into the Amundsen Bay (e.g., Dupont & Alley, 2005; Rignot et al., 2011).

**Figure 1:**
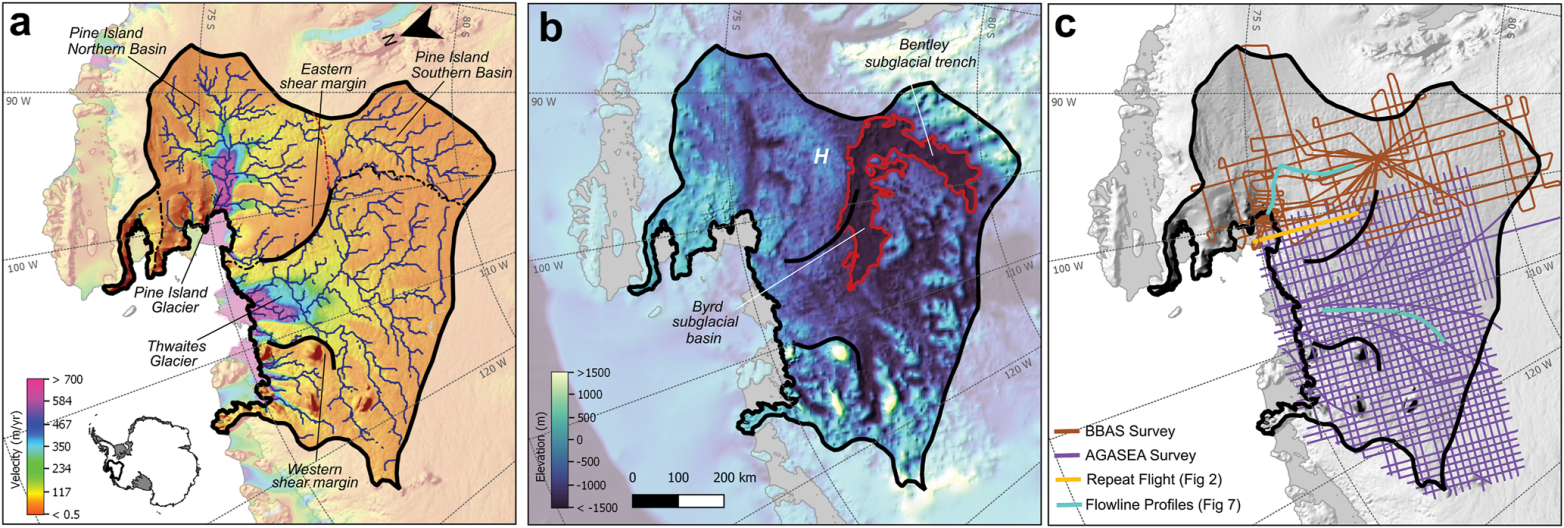
Site setting of the study region. (a) Pine Island and Thwaites Glaciers in the context of Synthetic Aperture Radar (SAR)/InSAR ice surface velocities (Rignot et al., 2014) and calculated subglacial drainage pathways (in dark blue; Chu, Creyts, & Bell, 2016; Chu, Schroedu, et al., 2016). (b) Bedmap2 bed elevation (Fretwell et al., 2013) with the Bentley Subglacial Trench (BST) and the Byrd Subglacial Basin (BSB) outlined in red; H marks the basal plateau identified by Vaughan et al. (2006). (c) Locations of the two airborne radar surveys collected during the BBAS (brown) and Airborne Geophysical Survey of the Amundsen Sea Embayment Antarctica (AGASEA; purple) campaigns. Gaps in the lines are locations where bed power picks failed to meet the quality thresholds described in Section 2.1. Example echogram in Figure 2 and flowline profiles in Figure 7 are highlighted in light orange and cyan lines, respectively.

Along the boundary of PIG and TG, there is a potential for dynamic interaction between these glaciers. The two glaciers are currently separated by the topographically unconfined eastern shear margin of TG (MacGregor et al., 2013) along which transitions in basal conditions potentially control the shear margin location (Schroeder, Grima, & Blankenship, 2016). In the ice sheet interior, the PIG drainage basin has also been previously delineated into northern and southern basins based on surface slope (Rignot et al., 2014; Vaughan et al., 2006). The more slowly draining southern basin overlays the Bentley Subglacial Trough (BST) and part of the Byrd Subglacial Basin (BSB); meanwhile the northern basin comprises the main ice trunk of PIG (Figures 1a and 1b). A ∼400‐m high basal plateau (marked H in Figure 1b) marks the separation between these two basins. Despite the presence of this plateau, the southern basin ice flows parallel to surface slope into PIG's northern basin instead of toward TG (Vaughan et al., 2006). This ice‐flow direction contrasts with subglacial flow pathways estimated with a water routing algorithm (Chu, Creyts, & Bell, 2016; Le Brocq et al., 2009; Wright et al., 2008), which predict a general drainage into TG (dark blue lines in Figure 1a). This range of potential cross‐glacier interactions between PIG and TG may shape the glaciers' dynamics, highlighting the need for a unified cross‐basin geophysical analysis.

To date, PIG and TG have only been comprehensively investigated independently using two airborne radar sounding surveys collected in the 2004–2005 austral field season (Holt et al., 2006; Vaughan et al., 2006; Figure 1c): (a) the Airborne Geophysical Survey of the Amundsen Sea Embayment Antarctica (AGASEA) that focused on the TG basin from the grounding line up to ∼400‐km inland (Holt et al., 2006); and (b) the British Antarctic Survey that targeted PIG (BBAS campaign), covering both the northern and southern basins. The radar sounding data from these surveys have produced major advances in understanding the basal topography (Fretwell et al., 2013), as well as basal conditions and ice/ocean processes (Bodart et al., 2021; Fudge et al., 2014; Goff et al., 2014; Grima et al., 2014; Karlsson et al., 2009, 2014; Muldoon et al., 2018; Schroeder et al., 2013). The purpose of this paper is to synthesize these surveys into a unified collection of radargrams and provide cross‐catchment estimates of basal reflectivity to investigate the potential interaction between the two glaciers. We will also leverage the published radar specularity content (Schroeder et al., 2013) and trailing bed echoes (Young et al., 2016) results at TG to produce a joint interpretation of radar reflectivity and scattering character. The integrated observations provide a critical 2004/2005 ASE‐wide baseline against which existing and future surveys can be compared to study the cross‐catchment interactions and the role of subglacial processes for controlling the dynamics and stability of this region.

## 2 Methods

**Figure 2:**
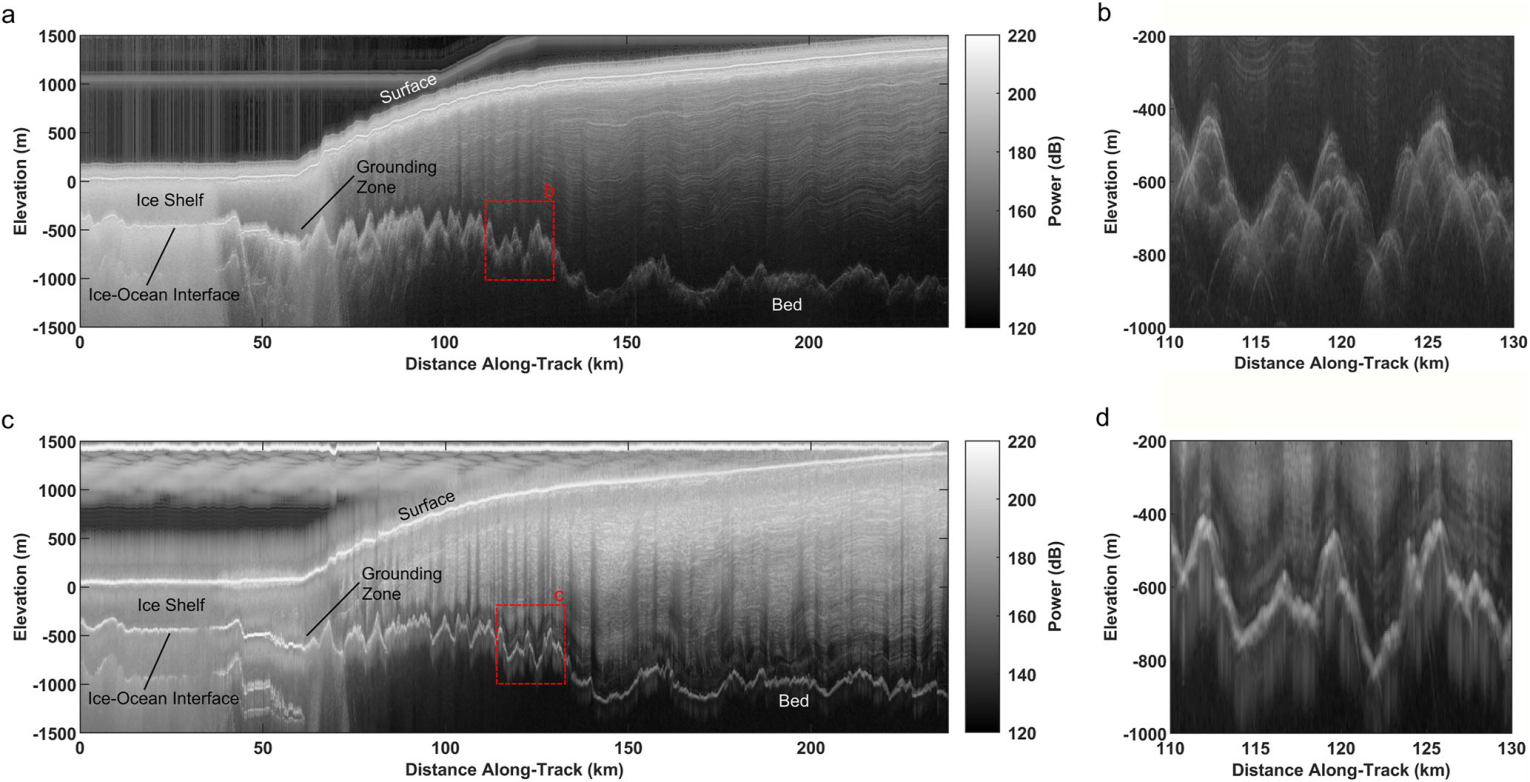
Depth‐corrected radargrams of the spatially coincident flight line (light orange line in Figure 1c) flown by both the (a) High Capability Airborne Radar Sounder (HiCARS; transect THW/SJB2/X83a, high gain channel) with an inset in panel (b) showing the bed echo character and (c) Polarimetric‐radar Airborne Science Instrument (PASIN; flight b21) radar with a similar bed inset in panel (d). Image grayscale represents relative echo power in decibels. Elevations are referenced to the WGS84 ellipsoid. Effects of the tighter cross‐track beam pattern and larger number of coherent summations for the PASIN system are visible in the form of shorter hyperbola tails on the bed echoes.

The AGASEA data were collected at TG using the 60 MHz center frequency, 15 MHz bandwidth High Capability Airborne Radar Sounder (HiCARS) system (Peters et al., 2007). The BBAS survey at PIG was conducted using the 150 MHz center frequency, 10 MHz bandwidth Polarimetric‐radar Airborne Science INstrument (PASIN) radar sounder (Corr et al., 2007; Vaughan et al., 2006). The impact of these different radar system parameters can be seen in the spatially coincident radargrams from each survey shown in Figure 2.

We analyze bed echoes extracted from unfocused Synthetic Aperture Radar (SAR) processed (Peters et al., 2007) radar sounding profiles for HiCARS (Figure 2a) and the incoherent, unfocused PASIN (Figure 2b) data. Note that because our primary focus is the deep ice, we only use the PASIN data acquired in the chirp‐acquisition mode and not the shallower‐sounding pulse data that were also collected simultaneously to the chirp. As a result, we eliminated 17 of the 32 original BBAS survey lines from this study due to the lack of chirp‐acquisition mode data. Both systems were flown on similar Twin Otter aircrafts with typical speeds of ∼70 m/s. PASIN operated at an effective pulse repetition frequency (PRF) of 312.5 Hz (Corr et al., 2007) and the data were processed with 70 coherent summations and 4 incoherent averages over range lines. HiCARS operated at an effective PRF of 200 Hz and used both high and low gain data channels to improve dynamic range. We process both channels with 10 coherent summations and 5 incoherent averages using HiCARS processing parameters that match those used in prior studies of unfocused AGASEA data (Schroeder, Blankenship, Young, & Quartini, 2014). To our knowledge, this is the first radiometric use of unfocused SAR processed data from the BBAS survey acquired in the chirp‐acquisition mode with processing parameters selected to minimize clutter at the higher system frequency. In total, we produce 1,081 50‐km long unfocused SAR radargrams from which we estimate englacial attenuation and basal reflectivity.

### 2.1 Bed Echo Power Extraction

To calculate basal reflectivity, we must first identify radar bed echo power. Bed echo power can be defined by either the peak or aggregated power with the latter corresponding to a summation over the echo envelope (Jordan et al., 2016; Oswald & Gogineni, 2008). While peak power is more sensitive to the contrast in basal material and thawed/frozen transition than aggregated power (e.g., Peters et al., 2005; Schroeder, Grima, & Blankenship, 2016), because PASIN and HiCARS have significantly different center frequencies, the use of peak power introduces variable losses from frequency‐dependent surface transmission (Schroeder, Grima, & Blankenship, 2016), volume scattering (Aglyamov et al., 2017), and basal roughness (Jordan et al., 2018; Peters et al., 2005) into the underlying bed reflectivity signal. As such, we opt to use aggregated power to suppress these effects, as aggregation recaptures some of the energy that is lost by scattering (Oswald & Gogineni, 2008).

We calculate aggregated power by summation in range over the bed echo power envelope truncated 10 dB below the peak (Jordan et al., 2018). For HiCARS data, we use power from the low gain channel so long as the peak power exceeds the noise floor by 10 dB. Otherwise, we use the high gain channel data adjusted for the power offset between channels. We also discard bed echoes that are <10 dB above the noise floor, those with envelopes that do not converge within twice the pulse‐limited footprint (Peters et al., 2005), echoes collected at regions with ice thickness <500 m, or traces where the sensor platform bearing changed >2°/km along‐track (MacGregor et al., 2015).

### 2.2 Echo Power Corrections

**Figure 3:**
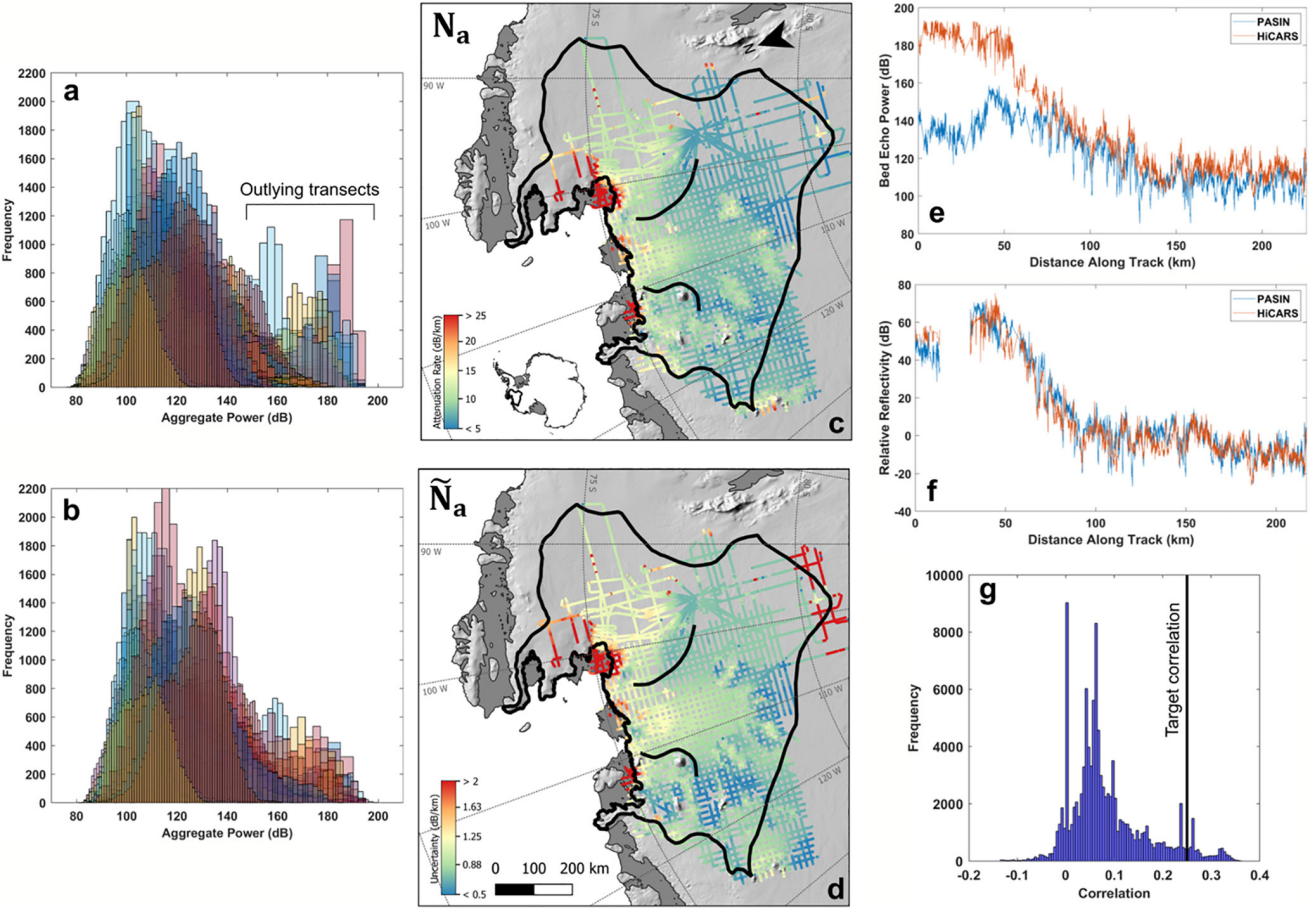
Histograms of geometrically corrected bed echo power (a) Before and (b) After cross‐leveling for all flight transects. (c) Estimated englacial attenuation rate for Amundsen Sea Embayment (ASE) and (d) Fitting uncertainty in the 2‐D empirical approach. A comparison of the (e) Initial bed power prior to any corrections and (f) The final reflectivity values along the common flight line (Figure 2) between Polarimetric‐radar Airborne Science Instrument (PASIN; blue) and High Capability Airborne Radar Sounder (HiCARS; orange). The significant difference in bed echo power between the PASIN and HiCARS between 0 and 75 km along‐track is due to the time dependent receiver gain in the PASIN data. (g) Histogram of plausible correlation targets, assuming that local minima contain wet till, and all other regions are frozen. The target correlation of 0.25 used for sensitivity testing is marked with the black line.

Next, we apply several corrections to the extracted bed power from both surveys to calculate basal reflectivity. These include corrections for power variations related to geometric spreading, radar system performance, englacial attenuation, and birefringence loss (Matsuoka et al., 2010). We calculate geometric spreading loss assuming an inverse square dependence on the sensor range (Haynes et al., 2018). We also assume that birefringence losses are small enough to be neglected (Matsuoka et al., 2012). To correct for the difference in radar system performance between HiCARS and PASIN, which includes the effect of fast‐time variable gain or receiver nonlinearity in the PASIN data, we fit an exponential function to the two‐way travel time from the surface to ice‐bed interface versus the difference in bed echo power between the overlapping HiCARS and PASIN transects. We use this best empirical fit to the data as a depth‐dependent power correction and apply it to all PASIN transects. We then follow Chu, Schroedu et al. (2016) to correct for other variations in radar system performance, including beam‐pattern gain at nadir, by applying a geostatistical leveling to the geometrically corrected bed echo power at flight intersections. We use a Huber penalty function with a 3‐dB corner to ensure the corrections are robust against outliers. Figures 3a and 3b show how the leveling process adjusts the set of transects toward a common mean and reduces the 95% range of bed echo power values from 69.9 to 61.4 dB.

### 2.3 Estimation of Empirical Attenuation

Englacial attenuation losses must also be corrected before basal reflectivity is calculated. We estimate attenuation rates by developing an empirical approach based on Schroeder, Seroussi, et al. (2016) that requires no assumptions on ice temperature or chemistry (Chu et al., 2018; MacGregor et al., 2016). We extend the previous one‐dimensional (1D) transect‐based adaptive attenuation‐fitting approach of Schroeder, Seroussi, et al. (2016) to two dimensions by selecting data from a circular window around each sample point. This 2D approach increases the local sample density when performing the correlation fits and is more suited to incorporate multiple intersecting BBAS flight‐tracks with variable orientations.

In the 2D attenuation‐rate fitting, we define a minimum initial radius of 20 km around each data point along a transect and calculate the correlation between ice thickness and attenuation‐corrected bed echo power for a range of attenuation rates within that radius. We then adaptively increase that radius up to a maximum radius of 500 km until three goodness of fit conditions are met: (a) the initial correlation between ice thickness and power is greater than 0.7, (b) the minimum correlation is <0.01, and (c) the fractional uncertainty in the estimated attenuation rate is <10% for a fitting correlation width of 0.1. (Note that these values are for an unsquared correlation coefficient). To identify the minimum radius that satisfied these conditions, a binary search is performed over the range of fitting radii. These steps are repeated for each transect, discarding points where the fitting conditions are not satisfied. The most commonly failed condition was the 10% uncertainty in attenuation rate. The resulting englacial attenuation rates and uncertainty are shown in Figures 3c and 3d, respectively.

## 3 Error Analysis

We examine the internal consistency of our empirical attenuation estimates for the PASIN and HiCARS data by comparing the initial bed power prior to any corrections (Figure 3e) to the final relative reflectivity along the repeat transect shown in Figure 2 (Figure 3f). This cross‐system check reveals significant initial echo strength differences of up to ∼50 dB between the two systems. After calibration, the final reflectivity estimates are in close agreement with an RMS difference of 7.4 dB and a squared correlation coefficient of 0.92 between the two flight lines, suggesting a successful cross‐calibration of the two systems.

### 3.1 Sensitivity Analysis of Attenuation Fitting

Our empirical attenuation correction assumes the observed correlation between bed echo power and ice thickness is entirely attributable to attenuation. However, in principle, the ideal correlation target for the attenuation fitting may not be zero (Matsuoka, 2011). Local minima in topography may collect water or contain saturated sediments, resulting in a small positive correlation between ice thickness and reflectivity. To investigate the effect of changes in reflectivity in our attenuation fitting, we conduct a sensitivity test to estimate the impact of a nonzero correlation target on the magnitude and spatial pattern of englacial attenuation.

To do this, we set the correlation target using six representative bed elevation profiles and assign absolute reflectivities of −6 ± 5 dB, consistent with wet till (Peters et al., 2005), in local minima deeper than 100 m and absolute reflectivities of −30 ± 5 dB, consistent with frozen till, to everywhere else. We then compute the true correlation between ice thickness and bed reflectivity over the fitting distances along those transects from the original attenuation calculation. Based on these correlations, we choose 0.25 as a conservative upper estimate of a physically reasonable target correlation (Figure 3f).

**Figure 4:**
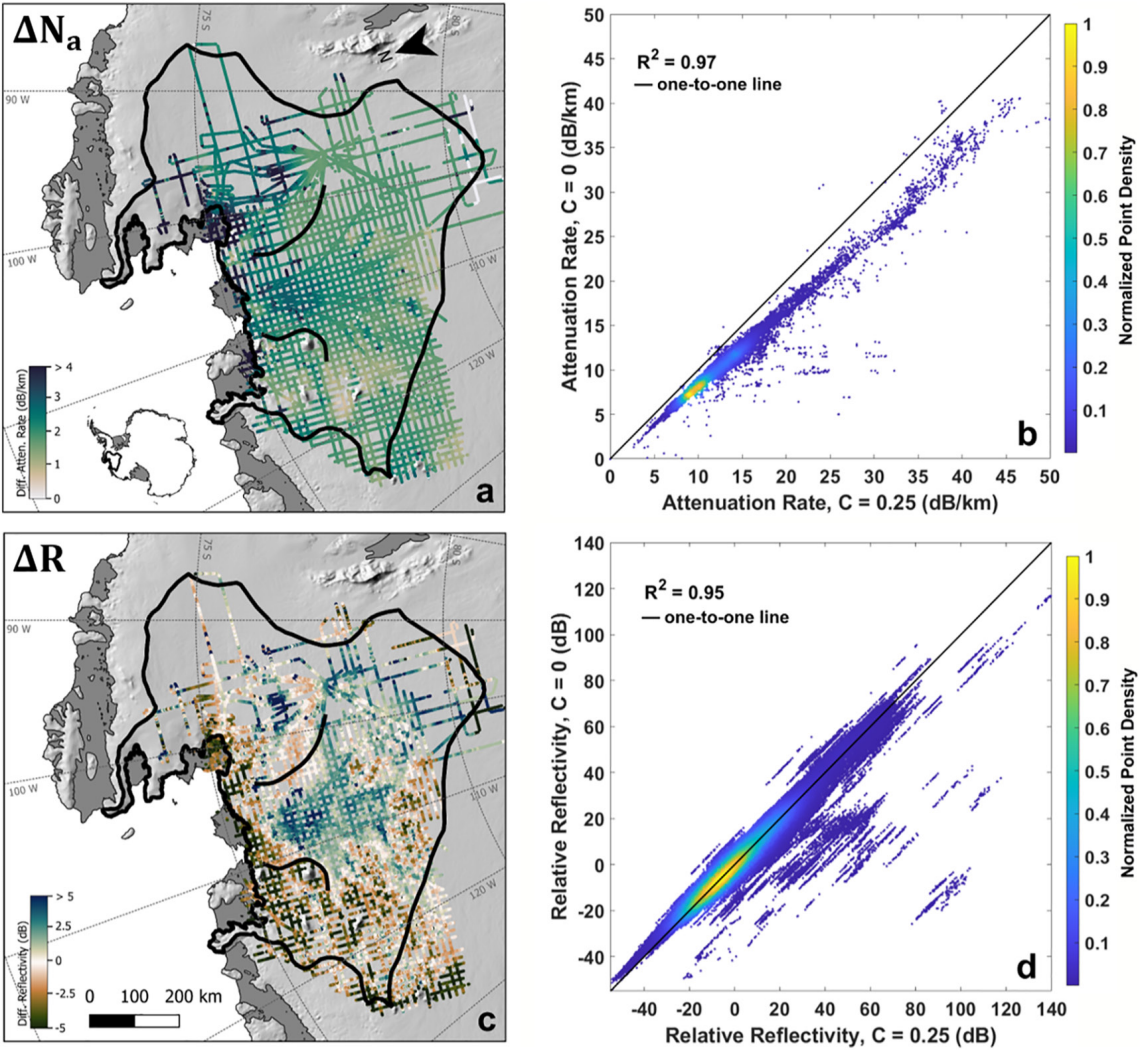
Difference between the (a) Attenuation rates and (c) Relative reflectivity estimated with target correlations of 0.25 and 0. Scatter plot of (b) Attenuation rates and (d) Reflectivity at the two target correlations. Brighter colors indicate a higher density of points, and the black line is the one‐to‐one line.

Figure 4a shows the difference between the attenuation rate estimates using a target correlation of 0.25 and a target correlation of zero. While the overall magnitude of the attenuation rate increases, our results illustrate minimal change to its spatial pattern, displaying a squared correlation coefficient of 0.97 between the two results (Figure 4b). We find a similarly high agreement between the spatial pattern of relative reflectivity using these attenuation rate estimates, with a correlation of 0.95 and an RMS difference of only 3.6 dB between the two results (Figures 4c and 4d). This sensitivity test suggests that the spatial patterns in both attenuation rate and relative reflectivity are largely insensitive to the choice of correlation target in the attenuation correction.

### 3.2 Uncertainty in Losses Related to Basal Roughness

**Figure 5:**
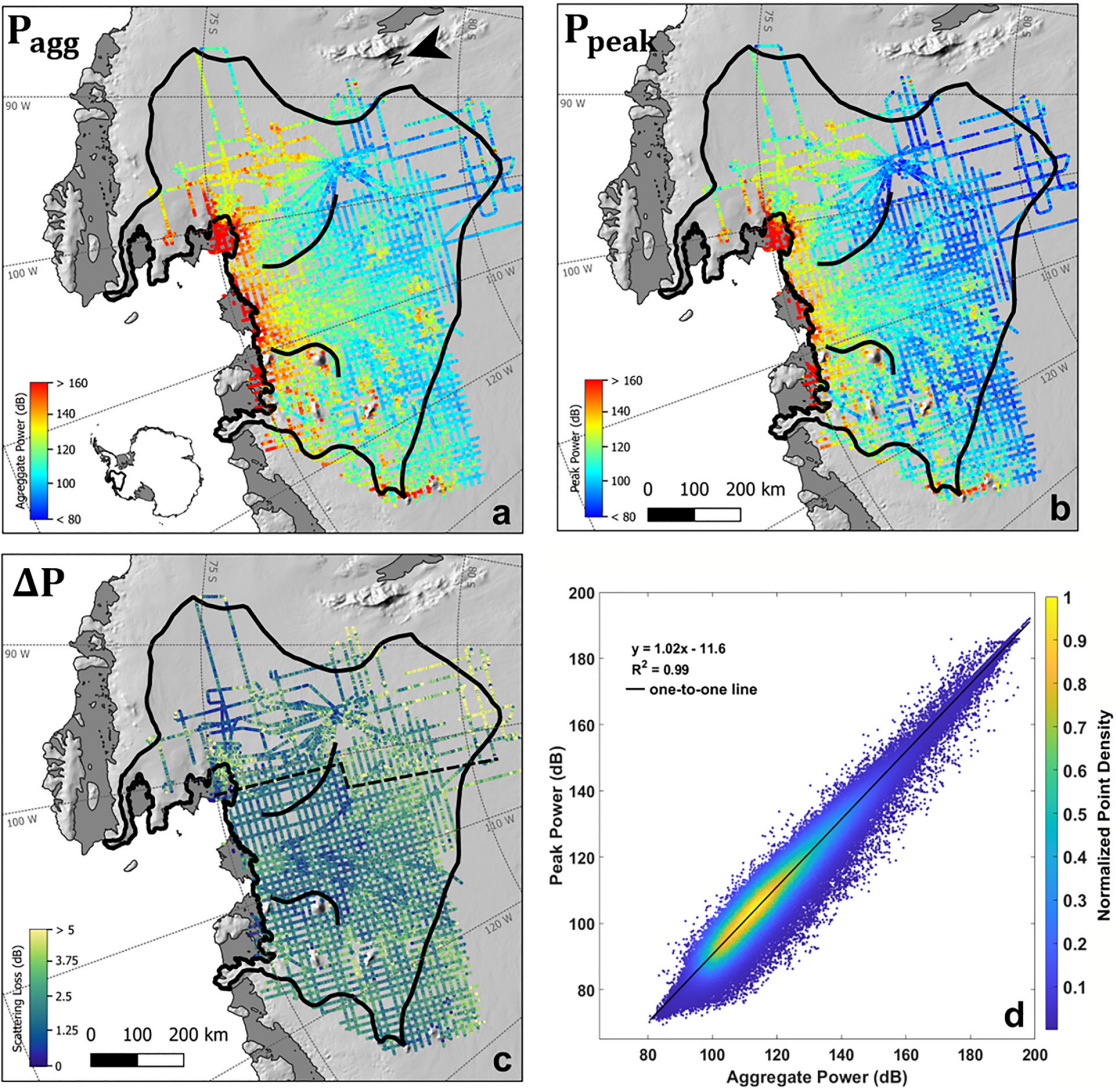
(a) Aggregated and (b) Peak geometrically corrected bed echo power for the combined Polarimetric‐radar Airborne Science Instrument (PASIN) and High Capability Airborne Radar Sounder (HiCARS) surveys. (c) Difference between aggregate and peak power where the dashed line marks the approximate boundary between the PASIN (east of line) and HiCARS (west of line) surveys. This metric shows strong frequency dependence between surveys and provides a rough proxy for the scattering‐loss uncertainty. (d) Correlation between aggregated and peak power and the black line is the one‐to‐one line.

In addition to the loss terms discussed above, scattering losses related to basal roughness at the scale of the radar wavelength can also induce uncertainty in basal reflectivities. We use the difference between the peak and aggregated bed echo power to estimate the effect of basal roughness. However, the absolute difference between aggregated power and peak power is not directly equal to the scattering loss. For any system with a finite range resolution, aggregated power will typically exceed peak power, even for an echo from an ideal specular interface. To account for this, we subtract from the raw difference between aggregated and peak power the theoretical difference between the aggregated (Figure 5a) and peak power (Figure 5b) of each system's impulse response, which also serves to correct for differences in system range resolution and sampling rate (Jordan et al., 2017). The approximated scattering loss (Figure 5c) illustrates that basal roughness has the greatest impact on basal reflectivity in regions surveyed by the PASIN system, which has a shorter wavelength and is, therefore, more sensitive to roughness.

### 3.3 Total Reflectivity Error

Finally, we combine all the error sources from scattering losses, uncertainties in englacial attenuation rates, and corrections for the difference in radar system parameters to estimate the total uncertainty in basal reflectivity. In particular, the uncertainty in attenuation rates is represented by either adding (or subtracting) the empirical fitting uncertainty (Figure 4b) from the mean attenuation rates (Figure 4a) to produce a maximum (or minimum) attenuation rate. We also include the mean cross‐over error of 4.2 dB as a conservative treatment of leveling effects. These errors are then propagated through the radar equation to produce different basal reflectivity. The maximum difference between these values represents the total error due to scattering losses and uncertainties in the corrections for cross‐system leveling and englacial attenuation losses.

**Figure 6:**
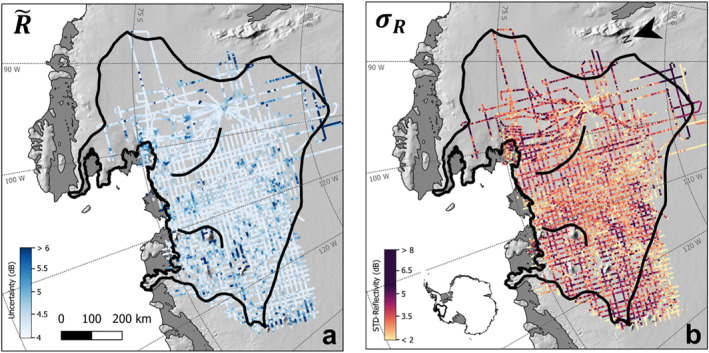
(a) Estimated uncertainty in relative reflectivity due to attenuation, roughness, and cross‐over errors. (b) Standard deviation of relative reflectivity calculated over a 5‐km moving window.

Figure 6a shows the resulting 95% uncertainty range in relative basal reflectivity is 4.2–5.6 dB with a mean value of 4.48 dB. This uncertainty is smaller than the combined absolute uncertainty in attenuation, scattering, and leveling, because the relative reflectivity is the spatial pattern of the reflectivity with the mean subtracted and is, therefore, insensitive to large‐scale shifts in the absolute value of the reflectivity. Figure 6b shows the local standard deviation of relative reflectivity over a 5‐km moving window. While this is not a measure of uncertainty per se, it provides a metric for how future measurements might vary locally from our results even absent of any temporal change in bed conditions.

## 4 Basal Reflectivity Results

**Figure 7:**
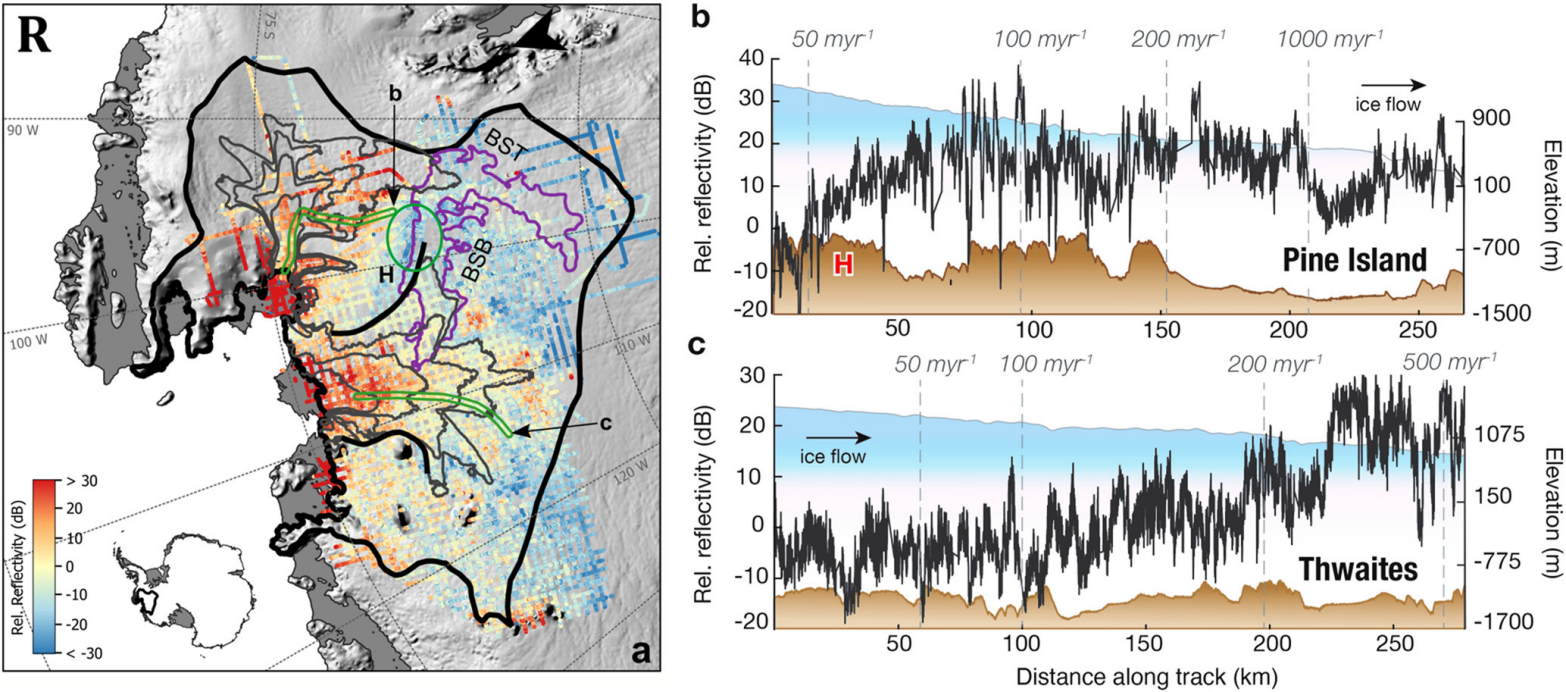
(a) Estimated relative reflectivity for the Amundsen Sea Embayment (ASE) region with ice velocity contours bed in gray marking 200, 150, 100, and 50 m yr^−1^. The green circle highlights the location of a frozen bed patch that could help to restrict ice in PIG's southern basin to branch into TG. (b and c) Approximately along‐flow profiles of relative reflectivity (dark gray) for (b) Thwaites Glacier and (c) Pine Island Glacier. Their locations are shown in the green outlines in panel (a) with the respective letter labels pointing to the start of each profile.

Figure 7a shows the relative basal reflectivity has a median value of −1.9 dB with a 95% confidence interval of −24.8 to +37.4 dB (Figure 7a). In both PIG and TG, we observe a general spatial alignment between higher relative reflectivity (>20 dB) and fast ice motion (>500 m yr^−1^) near the ice sheet margin (Figure 1a). We interpret these regions as water‐rich bed produced by elevated frictional and viscous heat dissipation associated with fast ice flow. In finer detail, however, PIG and TG show a variable spatial pattern in their observed basal reflectivities (Figures 7b and 7c).

### 4.1 Pine Island Glacier

In PIG, we observe a spatially extensive high reflectivity and thawed bed condition covering a vast area beneath the fast (>1,000 m yr^−1^) ice along the central ice trunk up to the slower (∼100–200 m yr^−1^) moving head of PIG's tributary 3 (Figure 1a). Despite the thick (>1,000 m) ice, the region north of BST displays atypically high relative reflectivities values exceeding 20 dB (Figure 7b). Similarly, in the region north of the central trunk, despite its sparse data coverage and relatively slow‐flowing ice, existing results show high relative reflectivities >20 dB near the Hudson Mountain volcanic region (Corr & Vaughan, 2008). In general, PIG's faster‐flowing northern basin has relative reflectivities above 0 dB (and as high as +37 dB). This contrasts with the slower‐moving southern basin in the BST, where we observe lower relative reflectivities of ∼0 to −22 dB. We interpret these lower values as an indication that the southern basin overrides a bed that is largely comprised of unsaturated and potential locally frozen materials. A previous study by Vaughan et al. (2006) identified a critical basal high (labeled “H” in Figure 1b) that connects the southern and northern basins of PIG. Our radar results display a relatively low relative reflectivities (−13 to −22 dB) patch, indicative of frozen bed conditions (Figure 7a, green circle), over and surrounding this basal high.

### 4.2 Thwaites Glacier

In TG, in contrast to PIG, we observe a more spatially variable reflectivity pattern. High relative reflectivity regions >20 dB are mainly confined in the retrograde bed within 100 km upstream of the grounding line (Figure 7c). As ice velocity decreases below 200 m yr^−1^, we observe a drastic reduction in relative reflectivities. To aid our discussions, we will divide the TG's basin into five zones from hereinafter: (a) “marginal zone” where TG's velocity exceeds 500 m yr^−1^; (b) “lower basin” where surface velocity is between 200 and 500 m yr^−1^; (c) “upper basin” where surface velocity is between 100 and 199 m yr^−1^; (d) “tributary head” with velocities range between 50 and 99 m yr^−1^; and (e) “ice sheet interior” where ice velocity drops below 50 m yr^−1^.

**Figure 8:**
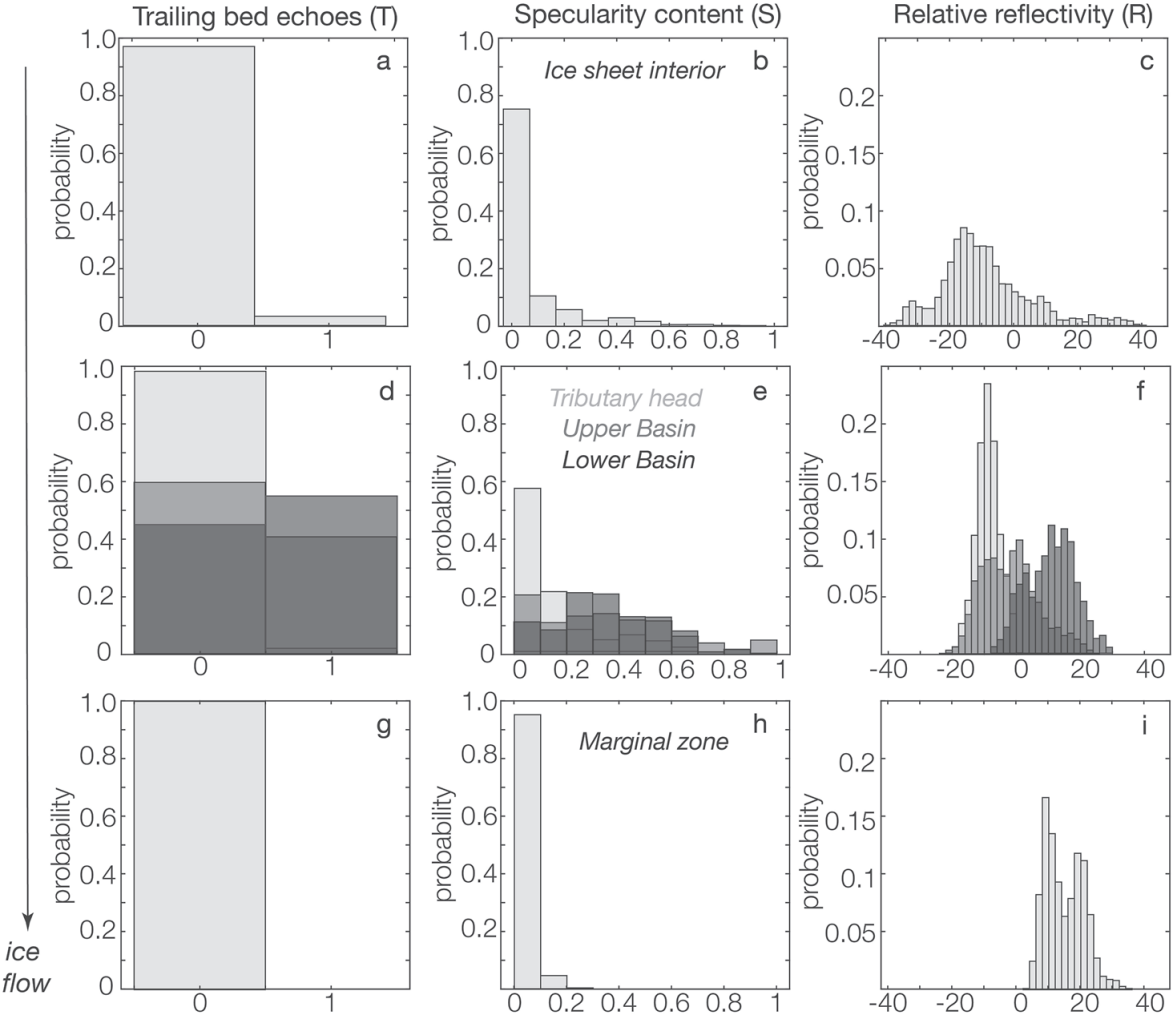
Along‐flow distribution of relative reflectivity (*R*), specularity content (*S*), and trailing bed echoes (*T*) at Thwaites Glacier. These values are from a radar profile that approximately aligns along ice flow direction (location is shown as B‐Bʹ in Figure 9a). The top row (a)–(c) Shows results from the “ice sheet interior zone” (<50 m yr^−1^); the middle row (d)–(f) Shows results from the “tributary head” zone (50–99 m yr^−1^) in light gray, “upper basin” zone (100–199 m yr^−1^) in moderate gray, and “lower basin” zone in dark gray (200–500 m yr^−1^); the bottom row (g)–(i) Shows the “marginal zone” (>500 m yr^−1^) results.

Figure 8c shows how relative reflectivities vary along these five zones of TG. In general, we observe an increase in relative reflectivities as we move closer to the central ice trunk of TG compared with the ice sheet interior. In the interior, we observe vast areas of low relative reflectivities (<−15 dB), indicative of potential frozen bed, as well as in the tributary head and ice sheet interior zones where mean reflectivities are ∼−8 dB. Further downstream, the mean relative reflectivity values increase from to −0.7 dB in the upper basin to ∼+14.6–15.3 dB in the lower basin and the marginal zone, respectively.

### 4.3 Along the Boundary of PIG and TW

Along the boundary of PIG and TW, a ∼400‐m high basal plateau (marked H in Figures 1b and 7a) marks the separation between the southern and northern basins of PIG (Vaughan et al., 2006). Vaughan et al. (2006) suggests that this basal plateau and the steep valley walls of PIG exert a strong topographic control on the current ice flow configuration of PIG and TW. From our reflectivity results (Figure 7a), we find that, in addition to topography, the current basal conditions surrounding the region of BST‐BSB also help to reinforce the present‐day flow configuration.

First, the basal plateau H is characterized with modest *R* of >0 to ∼+15 dB, suggesting that the bed is likely unfrozen and perhaps even contains some presence of basal water either as a water film or dilated till (Figure 7a). The potential presence of a sliding or deforming interface would help to ease the southern basin ice motion over the basal plateau *H*. Second, our results also show there is a ∼100‐km long, low *R* patch (green circle in Figure 7a) that presently separates PIG's southern basin and TG's ice in the BSB. The patch has *R* of ∼−8 to −20 dB (median *R* of −14 dB), indicating the bed interface is likely near or at a frozen condition (Peters et al., 2005). Although we eliminated some of the BBAS observations in this area based on the quality metrics described in Section 2.1, the remaining results surrounding the data gap generally show *R* of <−10 dB. Therefore, we believe the frozen patch is likely continuous across the data gap. The presence of a likely frozen bed or till with higher basal friction would have also made it more difficult for the southern basin ice in BST to branch westward into TG.

## 5 Discussion

### 5.1 Joint Reflectivity and Scattering Character Analysis

While high basal reflectivities are generally interpreted as an indication of subglacial water, a smooth bed or the presence of deformable sediments can complicate these interpretations. A joint analysis of reflectivity and radar scattering character, such as specularity content (Schroeder et al., 2013) or trailing bed echo (Young et al., 2016), can help to distinguish between the impacts of material contrast and feature geometry on radar power. The current PASIN data are unfocused and thus we cannot calculate their specularity content or trailing bed echoes. However, the HiCARS data in TG have been previously SAR‐focused (Schroeder et al., 2013; Young et al., 2016). We, therefore, jointly examine our newly generated basal reflectivities with these published estimates of radar scattering character to improve the overall basal interpretations at TG.

These scattering metrics include (a) specularity content (hereafter referred to as *S*), which describes the ratio of specular bed energy to the total of specular and diffuse power in the along‐track direction (Schroeder et al., 2013), and (b) cross‐track bed echo power (also known as trailing bed echoes; hereafter referred to as *T*), which describes the distribution of bed energy as a function of fast time as a proxy for off‐nadir scattering (Young et al., 2016). Higher *S* values indicate a smooth, mirror‐like interface, while lower values indicate a comparatively rough ice bed (e.g., Rutishauser et al., 2018; Schroeder et al., 2013). *T* is given by Young et al. (2016) as a binary value with 1 indicates the presence of off‐nadir scattering, whereas 0 represents an absence of scattering.

In principle, reflectivity (hereinafter referred to *R*) is more sensitive to material contrast, meanwhile, both *S* and *T* are more sensitive to changes in feature geometry, such as bed roughness (Oswald & Robin, 1973; Robin et al., 1969; Young et al., 2016) or subglacial water geometry (Schroeder et al., 2013). The main difference between *S* and *T* is that the former primarily responds to along‐track roughness, whereas *T* is mainly indicative of cross‐track scattering within the radar footprint (∼130‐m diameter for HiCARS). In other words, a bedform or drainage feature that is smooth along the direction of flight, but surrounded by rougher materials on either side would result in both high *S* and *T*. For the clearest indication for subglacial water, we look for signals with a combination of high *R*, high *S*, and low *T*, which suggest an isotropically smooth and flat ice‐bed interface.

**Figure 9:**
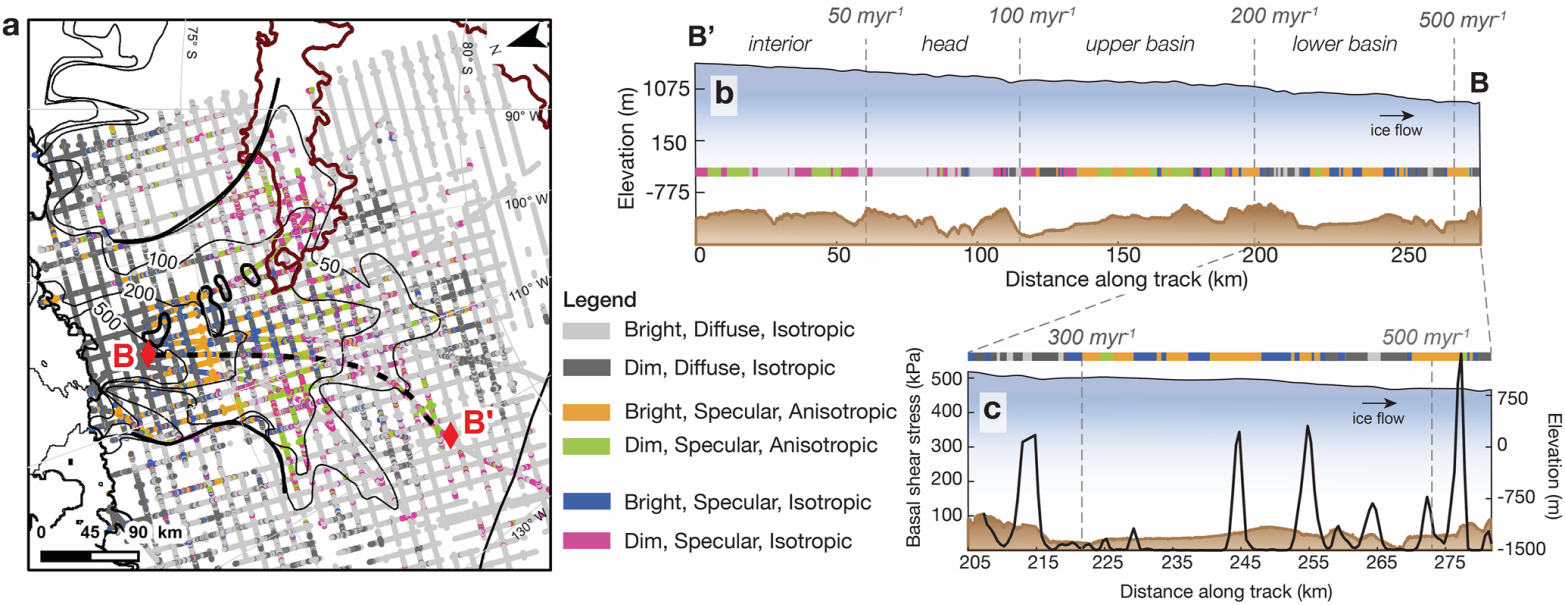
Updated classification map of basal conditions at Thwaites Glacier based on *R*, *S*, and *T*. In (a), the thin black lines show ice velocities contours at 50, 100, 200, 500 m yr^−1^ and the BSB‐BST outline in dark brown. The black dotted line shows the location of B‐Bʹ shown in panel (b). (b) Along‐flow profile (B‐Bʹ shown in panel a) illustrating the variations in radar classification along an ice flow line. (c) A zoom in profile in the lower basin zone where basal traction ribs (black lines) are modeled (Sergienko & Hindmarsh, 2013).

The final combined bed classification for TG based on *R*, *S*, and *T* is shown in Figures 8 and 9. Figure 8 illustrates how these metrics vary along a survey track that aligns approximately with ice flow (track location shown as B‐Bʹ on Figure 9a). Figure 9 shows their catchment‐scale distribution as an updated map previously published in Young et al. (2016). Following Young et al. (2016), we classified TG's bed into six broad categories: (a) bright (*R* > 0 dB), diffuse (*S* < 0.3), and isotropic (no trailing bed echoes), (b) dim (*R* < 0 dB), diffuse, and isotropic, (c) bright, specular (*S* > 0.3), and anisotropic (have trailing bed echoes), (d) dim, specular, and anisotropic; (e) bright, specular, and isotropic; and (f) dim (*R* < 0 dB), specular, and isotropic.

Together, these results suggest a diverse basal landscape and subglacial hydrologic conditions that evolve along‐flow with ice motion. In particular, Figure 8 displays a general trend of increasing *R*, *S*, and *T* as the glacier transitions from the ice sheet interior zone to the tributary head and upper and lower basins, indicating a progressively wetter ice‐bed interface with a basal roughness that displays stronger directionality along‐flow as ice velocity increases (Figures 8a–8f). However, as ice reaches the marginal zone (where surface velocity >500 m yr^−1^), we observe a reversal in *S* and *T* to lower values while *R* remains high, suggesting a shift in bed morphology back to a more isotropic roughness, similar to that observed in the ice sheet interior zone (Figures 8g–8i).

### 5.2 Implications for Large‐Scale Geomorphology and Subglacial Hydrology in TG

Although radar sounding alone only provides a general description of basal wetness and anisotropy in interface roughness, seismic reflection (e.g., Muto et al., 2019) and swath radar analyses (Holschuh et al., 2020) have been previously performed in TG. Furthermore, we have a decent understanding of glacial geomorphology based on studies on paleo‐ and modern ice streams (e.g., Dunlop & Clark, 2006; King et al., 2009; Smith & Murray, 2009). Informed by these earlier analyses, we can extend our radar interpretations on the spatial variations in geomorphology and subglacial hydrology in TG.

#### 5.2.1 Along‐Flow Evolution

A major feature we observed from the radar is the along‐flow increase in *R*, *S*, and *T* from TG's interior zone to the lower basin zone (Figure 8), which supports the existence of a generically rough bed in the ice interior that progressively smoothens approximately along‐flow down glacier. This pattern resembles the transition from ribbed moraines in glaciers' interior to more elongated, drumlin‐like bedforms with increasing ice motion that is commonly found in paleo landforms (Dunlop & Clark, 2006; Ely et al., 2016; Trommelen et al., 2014) and beneath modern ice streams in Antarctica (King et al., 2009; Smith & Murray, 2009). We observe this general along‐flow evolution in basal roughness across four out of the five tracks that align approximately with the ice flow direction., except for one track that crosses BSB.

#### 5.2.2 Upper Basin Zone

Another line of supporting observations we have is from seismic reflection (Muto et al., 2019) and swath radar analyses (Holschuh et al., 2020) previously conducted in TG's upper basin zone (i.e., which we define as the region where ice velocity is between 100 and 199 m yr^−1^). Both studies provide independent evidence for the existence of sediment‐mantled, crag‐and‐tail bedforms in TG's upper basin. In particular, the 40 km seismic reflection profile (Muto et al., 2019) across the crag‐and‐tails displays strong alternating variations in bed character, where consolidated till is found on the stoss side of the bed bumps and wetter, dilated till on the lee sides. We examine the radar tracks that are colocated with the seismic study and find similar broad‐scale alternations in bed reflectivity and scattering character across the crag‐and‐tail features (Figure 9b). At ∼100–200 km along this upper basin track, we observe a mosaic of lower *R*, higher *S* and *T* regions interspersed with higher *R* with similar *S* and *T*, suggesting that the crag‐and‐tails, in agreement with the seismic observations, likely consist of uniform bed material with comparable basal roughness but with a variable water content at its top interface. Based on our airborne coverage, we further deduce that crag‐and‐tail bedforms are highly prevalent, covering close to two‐thirds of TG's upper basin (Figure 9a).

#### 5.2.3 Lower Basin Zone

Interestingly, we observe similar banding variations in *R*, *S*, and *T* in the lower basin zone (defined as a region with a velocity between 200 and 500 m yr^−1^). Here, the radar displays alternating regions of high *R*, *S*, and *T* (similar to those observed in the crag‐and‐tail regions in the upper basin), now separated by areas with high *R*, high *S*, and low *T*, indicative of subglacial water ponding or saturated thin till layer with an isotropically smooth, mirror‐like interface. These results are consistent with a previous specularity study that suggests that the lower basin consists of a field of distributed subglacial canals surrounded by an area of deforming till (Schroeder, Blankenship, Young, Witus, & Anderson, 2014).

In addition, at ∼200–275 km along the lower basin track (Figure 9c), some of the radar reflectivity and scattering character variations appear to colocate with variations in modeled basal shear stress (Sergienko & Hindmarsh, 2013). Previous inversions of surface velocities of TG propose the potential existence of basal traction ribs (black line in Figure 9c) that may be related to varying subglacial hydrology. Based on visual inspection, we find some of the lower basal traction regions seem to occur in areas with high *R*, high *S*, and low *T*, which we infer to likely be regions of subglacial water ponding or distributed canals. While the presence of basal traction ribs is a matter of debate and they are notably absent in the latest model inversion (Morlighem et al., 2013), our radar results support the existence of a highly variable basal condition in the TG's lower basin that has the potential to produce varying basal drag. Future work combining radar observations with joint hydrology and stress balance models can further illuminate their spatial correspondence and causality.

#### 5.2.4 Marginal Zone

A final major feature we have highlighted is the reversal to low *S* and *T* while *R* remains high (>25 dB) in the marginal zone of TG (Figures 8g–8i). Schroeder, Blankenship, Young, Witus, & Anderson (2014) previously interpreted the margins of TG consist of a field of subglacial channels based on this low *S* character. While the presence of subglacial channels is consistent with hydrology modeling (Hager et al., 2020) and ice shelf channels observations (Alley et al., 2016; Le Brocq et al., 2013), we now believe that subglacial channels alone do not produce the observed low *S* and *T* across a large, hundreds of km square region. This is because, given the 15 km spacing of radar tracks and the fact that channels tend to concentrate toward the glacier margin, the chances of missing the channels are very high. Instead, our new interpretations based on the updated *R*, *S*, and *T* results is that the marginal zone of TG is composed of a generically rough bed with meandering subglacial channels that produce locally high *R*. In terms of radar sounding, this updated view means that we now believe that the low *S* is not a product of channel shape and its rougher geometry relative to distributed canals (Schroeder, Blankenship, Young, Witus, & Anderson, 2014), but instead the low *S* is more representative of the surrounding basal roughness and in contrast to a distributed system, subglacial channels can transport significant water without raising the background specularity content.

## 6 Conclusions

We analyze a total of 1,081 unfocused SAR radargrams, covering ∼54,000 line‐km from two simultaneous airborne radar surveys of PIG and TG to produce estimates of englacial attenuation and basal reflectivity. These results provide a cross‐system, cross‐catchment 2004–2005 baseline against which other geophysical studies can be compared. Our analysis of the sensitivity of the relative reflectivity patterns to breakdowns in the assumption of uncorrelated ice thickness and reflectivity highlights the interpretive robustness of relative rather than absolute reflectivity patterns. We also combine the relative reflectivity estimates with previously published results on specularity content and trailing bed echoes at TG. Together, these results display several noteworthy subglacial features across TG:

1. The ice sheet interior of TG is marked by a generically rough bed (at the 130‐m radar footprint scale) that is largely unsaturated and maybe frozen in localized places.
2. The glacier bed progressively becomes wetter and smoothens along‐flow as ice velocity increases from <50 m yr^−1^ in the interior to 500 m yr^−1^ in the lower basin zone. We interpret this evolution to be the along‐flow transition of ribbed moraines (or other similar transverse bedforms) to drumlin‐like features.
3. The upper and lower basin zones of TG, that is, the region where surface velocity is between 100 and 500 m yr^−1^, are characterized by a highly heterogeneous bed with distinct bands of varying basal wetness and roughness. In the upper basin, we believe these bands of varying radar reflectivity and scattering character likely correspond to crag‐and‐tails and their changing dilated/consolidated till cover previously observed by seismic and swath radar studies. In the lower basin, we observe a clear indication of meltwater ponding, which likely reflects the presence of distributed canals in an area of deformable subglacial tills.
4. The bed at the marginal zone (where velocity >500 m yr^−1^) is likely characterized by a system of subglacial channels cutting across an isotropically rough region. The presence of channels can transport a significant amount of water without raising the background specularity content.

Finally, with our new cross‐system, cross‐catchment reflectivities, we also identify a potential frozen bed patch in the Byrd Subglacial Basin that currently separates the ice mass of PIG's southern basin and the eastern tributary of TG. Overall, our study suggests that combining airborne radar analysis with ground validation from seismic and geomorphological observations provides a powerful toolset for understanding variations in subglacial hydrology and roughness and how they impact cross‐catchment interactions of ice streams and glaciers.

## Data Availability

All of the processed radargrams and derived parameters from this study have been posted on the USAP‐DC at https://doi.org/10.15784/601436.
